# A Case of Sublingual Gland Hypertrophy in the Setting of Submandibular Gland Aplasia Presenting as a Neck Mass

**DOI:** 10.1155/2024/8610465

**Published:** 2024-06-17

**Authors:** Colten Wolf, Richard Hubbell

**Affiliations:** Loyola University Medical Center, Department of Otolaryngology, Maywood, IL, USA

## Abstract

**Background:**

Absence or aplasia of the major salivary glands is an uncommon diagnosis and is often associated with other congenital abnormalities. Agenesis of a single submandibular gland, however, is an even more rare phenomenon and can be associated with hypertrophy of other salivary glands.

**Methods:**

A 48-year-old female presented to the clinic with a left-sided neck mass below her mandible. Workup including a CT scan showed an absent left-sided submandibular gland and an enlarged sublingual gland protruding through the mylohyoid muscle.

**Results:**

The patient underwent a transoral resection of the mass with subsequent resolution of the mass. The pathology returned as normal salivary gland tissue.

**Conclusions:**

Sublingual gland hypertrophy is a very uncommon presentation for a patient with a neck mass. This situation can arise in the setting of submandibular gland aplasia and compensatory hypertrophy of other salivary glands.

## 1. Introduction

The submandibular glands arise during the 6th week of development and the sublingual glands arise during the 9th week of fetal embryogenesis. These glands originate from epithelial buds in the floor of the mouth and become well differentiated by the 16th week of development [1, 2]. The first case of salivary gland aplasia was published in the late 19th century by Gruber during a cadaveric dissection [3]. Since this time, there have been many reports of submandibular gland aplasia and this anomaly is often associated with other congenital abnormalities such as cleft lip, cleft palate, or other syndromes such as Treacher Collins [4, 5]. Agenesis of a single submandibular gland without the presence of other head and neck developmental anomalies is less common and can be seen with compensatory hypertrophy of other glands [6].

In this case, we will describe a patient with agenesis of a single submandibular gland with a compensatory hypertrophy of the ipsilateral sublingual gland presenting as an asymptomatic neck mass.

## 2. Case

A 48-year-old female with a past medical history of asthma and acid reflux presented to the clinic with an eight-year history of swelling of her left neck below her mandible. She had no pain and the fullness below her mandible was her only symptom. She had been previously seen this year earlier and was diagnosed with sialadenitis. The swelling persisted despite conservative management leading her to seek a reevaluation.

**Figure 1:**
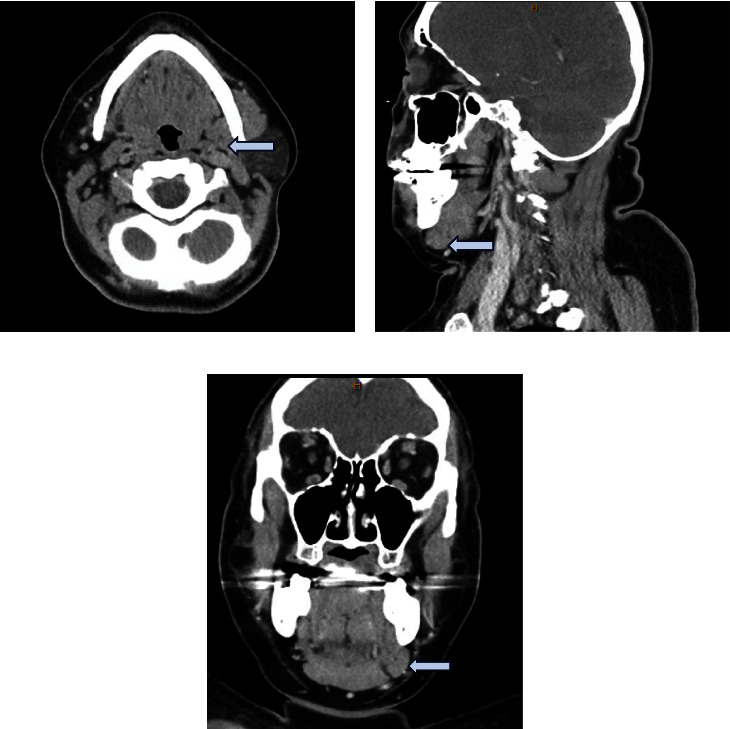
Protrusion of the sublingual gland through the mylohyoid (axial (a), sagittal (b), and coronal (c)).

**Figure 2:**
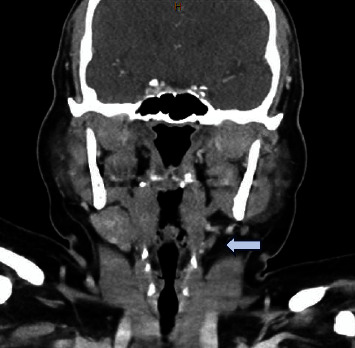
Absent submandibular gland on the left side.

**Figure 3:**
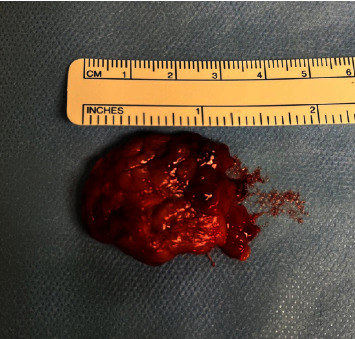
3.5 cm sublingual gland.

On exam, she had fullness in her left neck below the anterior body of her mandible. A bimanual exam also revealed fullness in the oral cavity. No other abnormalities were noted on the exam and she had no lesions within her oral cavity. A CT neck with contrast showed an absent submandibular gland on the left side with an enlarged left-side sublingual gland. The patient was referred to our clinic with the CT already completed, which is why an ultrasound was not ordered first. The left sublingual gland extended through the mylohyoid boutonniere (Figures 1 and 2). Given the persistence for multiple months, a decision was made to go to the OR for excision rather than a biopsy of the mass. The procedure was performed under general anesthesia. She was taken for a transoral excision of the left sublingual gland with subsequent resolution of her symptoms (Figure 3). The procedure was performed by incising through the mucosa of the floor of the mouth revealing the large sublingual gland extending down into the neck. The pathology returned as normal sublingual salivary gland tissue.

A differential diagnosis for this patient with an asymptomatic neck mass is very broad. Diagnosis to consider for this patient includes congenital anomalies, inflammatory or infectious processes, benign neoplasms, and malignant neoplasms either primary or metastatic to a lymph node from another head and neck malignancy. Imaging is a crucial tool in determining the etiology of the mass and revealed the abnormal salivary gland development in our patient.

## 3. Discussion

Submandibular gland aplasia is an uncommon condition that is usually associated with other developmental anomalies. No such anomalies were present in our patient.

Many patients with submandibular gland aplasia present without symptoms; however, this is highly variable. When symptoms are present, patients may notice xerostomia due to decreased salivary gland function leading to other issues such as dental caries, dysphagia, or abnormal taste [4–7]. Many of these patients will not have symptoms of dry mouth due to a compensatory increase in saliva production from the other glands. Patients with more symptomatic xerostomia often times have aplasia of multiple salivary glands. It may be that the asymptomatic presentation of many patients has led to significant underreporting of this condition.

There are several reports of submandibular gland aplasia reported in the literature. Most of these patients are asymptomatic, and a neck mass is a rare presenting symptom. Most cases involving a neck mass generally find the mass on the opposite side of the aplastic submandibular gland due to hypertrophy of the contralateral submandibular gland [7]. This can have clinical importance in patients with a history of oral cavity carcinoma. There have been cases reported of patients with a history of oral cavity carcinoma with an enlarged mass in level 1B found on surveillance imaging who had submandibular gland hypertrophy in the setting of contralateral gland aplasia [8]. Biopsy or FNA of neck masses is a critical step in the workup of these lesions. Due to the chronicity of this mass and patient preference, we elected for an excisional biopsy as opposed to FNA. There are two other case reports that describe cases of sublingual gland hypertrophy in the setting of submandibular gland aplasia [9, 10]. However, both of these reports describe cases of asymptomatic patients where the mass was incidentally identified on imaging as opposed to the patient presenting with a neck mass as in our patient.

The diagnosis of submandibular gland aplasia and compensatory hypertrophy of other glands is made via imaging, which include CT, MRI, or ultrasound. Ultrasound may be a good initial test to image the salivary glands, but CT and MRI are often needed to view deeper structures. Imaging is important to distinguish a mass in the submandibular area from other causes which can be benign, malignant, congenital, or acquired masses.

## 4. Conclusion

Sublingual gland hypertrophy is a very uncommon presentation for a patient with a neck mass. This situation can arise in the setting of submandibular gland aplasia with compensatory hypertrophy of other salivary glands. It is important to consider this rare condition in the differential diagnosis as a rare cause of a patient with a neck mass.

## Data Availability

The data used to support the findings of this study are available from the corresponding author upon reasonable request.
